# Towards improved uptake of malaria chemoprophylaxis among West African travellers: identification of behavioural determinants

**DOI:** 10.1186/1475-2875-12-360

**Published:** 2013-10-10

**Authors:** Rosanne W Wieten, Janneke Harting, Pieter M Biemond, Martin P Grobusch, Michèle van Vugt

**Affiliations:** 1 Center of Tropical Medicine and Travel Medicine, Department of Infectious Diseases, Academic Medical Center, University of Amsterdam, Amsterdam, the Netherlands; 2 Department of Behavioral Sciences, Academic Medical Center, University of Amsterdam, Amsterdam, the Netherlands

**Keywords:** Malaria, Travellers, Chemoprophylaxis, Behavioural determinants

## Abstract

**Background:**

Malaria is a potentially lethal illness for which preventive measures are not optimally used among all travellers. Travellers visiting friends and relatives in their country of origin (VFRs) are known to use chemoprophylaxis less consistently compared to tourist travellers. In this study, factors explaining the low use of chemoprophylaxis were pursued to contribute to improving uptake of preventive measures among VFRs.

**Methods:**

Following in-depth interviews with Ghanaians living in Amsterdam, a questionnaire was developed to assess which behavioural determinants were related to taking preventive measures. The questionnaire was administered at gates of departing flights from Schiphol International Airport, Amsterdam (the Netherlands) to Kotoka International Airport, Accra (Ghana).

**Results:**

In total, 154 questionnaires were eligible for analysis. Chemoprophylaxis had been started by 83 (53.9%) and bought by 93 (60.4%) travellers. Pre-travel advice had been obtained by 104 (67.5%) travellers. Those who attended the pre-travel clinic and those who incorrectly thought they had been vaccinated against malaria were more likely to use preventive measures. Young-, business- and long-term travellers, those who had experienced malaria, and those who thought curing malaria was easier than taking preventive tablets were less likely to use preventive measures.

**Conclusion:**

Almost half of the VFRs travelling to West Africa had not started chemoprophylaxis; therefore, there is room for improvement. Risk reduction strategies could aim at improving attendance to travel clinics and focus on young-, business and long term travellers and VFRs who have experienced malaria during consultation. Risk reduction strategies should focus on improving self-efficacy and conceptions of response efficacy, including social environment to aim at creating the positive social context needed.

## Background

In spite of numerous attempts to control malaria, it remains one of the most important life-threatening infectious diseases worldwide. Annually, an estimated 490,000 to 836,000 people die from malaria [1,2]. In industrialized countries, infections are reported among travellers returning from endemic areas, with currently approximately 6,000 reported cases in Europe [3] and 1,700 reported cases in the US, Canada and Australia [4-6]. These numbers are collected through passive surveillance and therefore likely to be underestimated. The heaviest burden lies in West Africa and 42 to 68% of imported infections are acquired here [7-9]. In the Netherlands around 240 malaria cases have been reported annually from 2007–2011 [10]. Travellers to Ghana contributed most (23%) cases of imported malaria, followed by Nigeria (14%), The Gambia (7%), Guinea (5%), and Uganda (4%).

Many travellers visiting African countries are visiting friends and relatives (VFRs). These VFRs are less likely to seek pre-travel health advice and have a tendency to use preventive measures less often compared to travellers with other travel purposes, such as tourism [11-14]. VFRs heading to West African countries have a high risk of contracting malaria [11,12,15,16]. A majority of these malaria cases could have been prevented with appropriate malaria preventive measures, such as bed nets, mosquito repellents and chemoprophylaxis. Therefore, a change in behaviour by VFRs travelling to West Africa seems to be required.

Successful behaviour change interventions are dependent on the ability to influence determinants affecting behaviour. Several determinants have been identified as predictors for taking prophylactic measures among VFRs. Basic knowledge has been associated with using prophylactic measures [17]. Incorrect knowledge of malaria has been also reported [18-20]. Accurate risk perception (people’s subjective assessment of the risk of malaria) has been described [18,19,21], and associated with using prophylactic measures [17,22]. Attitudes towards using prophylaxis (the degree to which one is in favour or against personally using preventive measures) have also been found to influence use of preventive measures [17,18,20-22]. However, determinants that can be influenced to improve uptake of malaria prophylaxis among VFRs travelling to West Africa are still to be identified.

The aim of this research was to quantitatively assess which determinants explain uptake of malaria chemoprophylaxis (starting and buying of chemoprophylaxis and obtaining pre-travel advice) among West African VFRs.

## Methods

For this cross-sectional observational study, questionnaires were administered to VFRs travelling to West Africa. A five- to ten-minute questionnaire was constructed and administered to travellers waiting at Schiphol International Airport, Amsterdam, at the boarding gates of flights to Kotoka International Airport, Accra (Ghana). Two researchers (PB and RWW) approached, at random, as many persons as possible during the waiting time at the gates for each flight (usually around two hours), and asked whether they were willing to answer the questions that would take five to ten minutes. Interviews took place during a period of 11 days in July 2012. The software program QuickTapSurvey (TabbleDabble, Toronto, Canada, 2011) was installed on mobile handheld computers to collect and store data.

Effective chemoprophylaxis (e g, atovaquone/proguanil, doxycycline or mefloquine) should be started on the day of departure or earlier. In this study, three outcome measures for uptake of chemoprophylaxis were used: (1) whether chemoprophylaxis had been started on the day of or before departure; (2) whether chemoprophylaxis had been bought; and, (3) whether pre-travel advice had been obtained. These behaviours were investigated with questions that could be answered with yes or no. The target population consisted of travellers aged >17 years. The analysis included travellers who had been living outside of West Africa for at least one year and were born in West Africa or of whom at least one parent was born in West Africa.

Group size was calculated according to the number of predictors in the logistic regression model. The largest number of participants would be needed if 50% of travellers had started chemoprophylaxis. If at least seven determinants would be added in the model and 10 participants per determinant would be required [23], 10 × 7 × 2 = 140 participants would be the absolute minimum.

### Variables

In order to assess personal variables, data on demographic details (sex, age, country of residence, country of birth, date of departure from country of birth), travel details (travel purpose, destination and duration of travel) and previous experience (whether travellers had contracted malaria before) were collected.

Education was not included because among the international population, many different schooling systems exist and this was not a uniform measure of knowledge.

**Figure 1:**
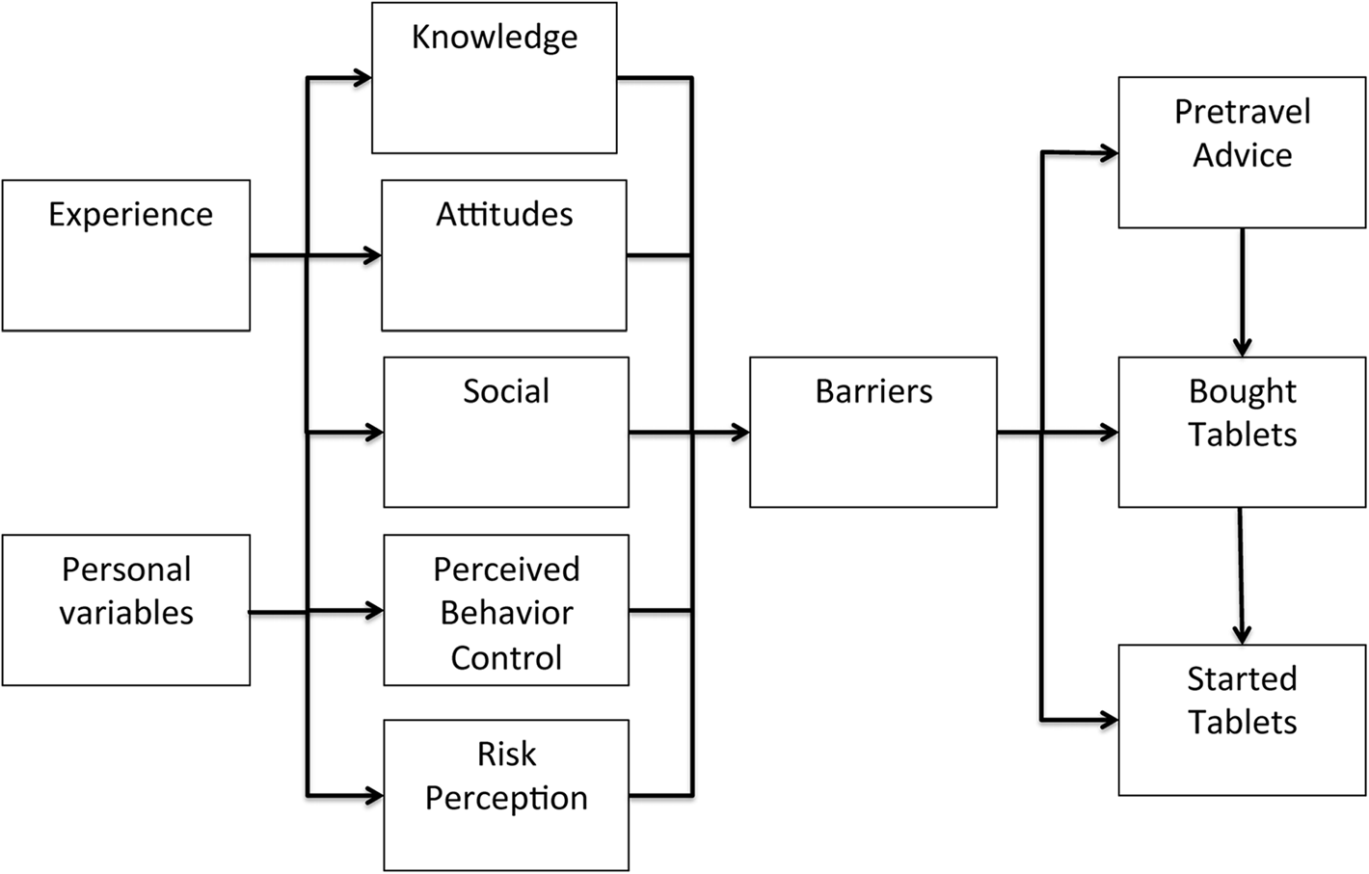
**Behavioural model for obtaining pre-travel advice, buying anti-malarial tablets and starting anti-malarial tablets.** Experience includes experience of disease, of the use of chemoprophylaxis and previous travel. Personality variables include demographics and travel details. Social includes social support and model behaviour. Determinants were structured based on three behavioural models. The Theory of Planned Behaviour (TPB) includes external variables (demographics, personality traits and environmental influences), attitudes (whether people regard a given behaviour positively or negatively), subjective norms (what the social environment thinks about the behaviour and how it acts) and perceived behaviour control (PBC) (expected personal performance of behaviour) as determinants. These determinants influence intention to perform behaviour, and intention predicts whether behaviour is performed. In the Health Belief Model (HBM), motivation to perform behaviour, perceived health threat and perceived reduction of this threat determine whether a given behaviour is performed. The Protection Motivation Theory (PMT) includes perceived severity of a threatening event, vulnerability of individuals (the chance that the health threat will occur), efficacy of recommended preventive behaviour and self-efficacy (defined as PBC in the TPB). This theory includes previous behaviour as an additional determinant.

To select and specify determinants to be included in the questionnaire, eight in-depth interviews with Ghanaians of various socio-economic backgrounds living in Amsterdam were conducted. Topics discussed were malaria, taking prophylactic measures, and making travel preparations. The determinants potentially influencing the use of chemoprophylaxis were identified and arranged in a model (Figure 1) based on three behavioural theories; the Theory of Planned Behaviour (TPB) [24], the Health Belief Model (HBM) [25], and the Protection Motivation Theory (PMT) [26]. The questions to assess behavioural determinants are shown in the Additional file 1. Statistical analyses were performed using PASWstatistics19 (IBM, Chicago, IL, USA).

### Analysis

In order to assess the relationship between the three prophylactic behaviours and VFRs’ demographics, previous malaria experience and travel details, univariate analyses were carried out. For binomial outcomes chi-square tests; for normally distributed continuous outcomes Student’s t tests; and for non-parametric continuous outcomes, Mann Whitney U tests were used.

On theoretical grounds, reliability analyses (assessment of internal consistency) were applied in order to construct determinant scales using the Cronbach’s alpha statistic. This resulted in low values of internal consistency [27]: 0.49 for knowledge, 0.17 for attitudes, 0.23 for risk perception, 0.38 for social influence, 0.36 for perceived behavioural control, and −0.42 for barriers. Therefore, the questions were analysed as separate variables.

To determine which determinants explained malaria prophylactic behaviours, a block-wise logistic regression analysis for each of the three outcome variables was carried out. Due to the small sample size, the number of determinants (independent variables) included was restricted. For this reason, variables were selected as described below.

The first block included independent variables that had a significant influence (p value <0.05) on the outcome measure in univariate analyses. Two of these variables (age and time of departure from country of birth) were highly correlated with each other (Pearson’s correlation -.718, p = <0.01). In a clinical setting, risk groups based on age were regarded to be easier to work with, therefore year of departure from country of birth was excluded from the logistic regression model.

The second block included determinants in the regression model that significantly correlated (p-value of <0.10) with the dependent variables in a bivariate Pearson’s correlation analysis (Additional file 2). The third block included previous use of chemoprophylaxis if it correlated with the outcome measure (Pearson’s correlation <0.10, Additional file 2).

## Results

### Participants

There was an approximate rejection rate of 10%. Out of a total of 164 participants recruited, 154 met both inclusion and exclusion criteria. The completion rate was 100%. As shown in Table 1, 81/154 (52.6%) participants were male. The mean age was 40.8 (SD 12.7); 82/154 (53.2%) were living in North or Central America and 72/154 (46.8%) in Europe. The main travel purpose was family affairs (134/154; 87.0%).

**Table 1:** Personal variables of 154 VFR travellers to Ghana

	**Total N = 154**	**%**	**Started CP N = 83**	**%**	**Not started CP N = 71**	**%**	**RR [CI]**	**p-value**
Sex, N (%) ^a^								
Male	81	(52.6)	38	(45.8)	43	(60.6)	0.76 [0.56-1.02]	0.067
Age, N (%) ^b^								
Mean	40.8		42.6		38.8			**0.033**
SD	12.7		12.8		12.4			
Missing N (%)	9	(5.8)						
Country of residence ^a^							1.22 [0.91-1.64]	
North and Central America, N (%)	82	(53.2)	40	(48.2)	42	(59.2)		0.17
Europe, N (%)	72	(46.8)	43	(51.8)	29	(40.8)		
Country of birth in West Africa^c^							0.76 [0.51-1.13]	
Ghana, N (%)	133	(86.4)	71	(85.5)	62	(87.3)		0.246
Other West African country^1^, N (%)	8	(5.2)	3		5			
				(3.6)		(7.0)		
Outside West Africa, N (%)	13	(8.4)	9	(10.8)	4	(5.6)		
Departure country of birth (Y) ^b^								
Median	1997		1993		2001			
Interquartile range	1990-2002		1989-1999		1992-2007			**<0.001**
Traveller VFRs, N (%)	13	(8.4)						
Previous visit, N (%)^a^	139	(90.3)	78	(94.0)	61	(90.0)	1.35 [0.68-2.67]	0.09
Had malaria before ^a^								
Yes, N (%)	85	(55.2)	37	(50.0)	48	(71.6)	0.66 [0.48-0.89]	**<0.001**
No, N (%)	56	(36.4)	37		19	(28.4)		
Missing, N (%)	13	(8.4)		(50.0)				
Travel purpose, family affairs ^a^								
Family affairs^2^, N (%)	134	(87.0)	78	(94.0)	56	(78.9)	2.33 [1.08-5.04]	**0.005**
Other^3^, N (%)	20	(13.0)	5	(6.0)	15	(21.1)		
Period West Africa >6 weeks ^a^								
<6 weeks, N (%)	119	(77.3)	76	(91.6)	43	(63.2)	0.34 [0.18-0.67]	**<0.001**
>6 weeks, N (%)	32	(20.8)	7		25			
Missing, N (%)	3	(1.9)		(8.4)		(36.8)		

CP = chemoprophylaxis, RR = Relative risk, ^a^ p-value calculated using the chi-square test; ^b^ p-value calculated using the Mann–Whitney U test; ^c^ p-value calculated comparing those born in West Africa to those born outside West-Africa using chi square test.

Significant differences are indicated in boldface.

^1^ Other countries of birth in West Africa were: Nigeria (three), Liberia (two), Cote d’Ivoire (one), Togo (one) and Sierra Leone (one).

^2^ Family affairs comprise VFR travel, funerals and weddings.

^3^ Other travel purposes are business (ten), holiday (four) and other (six).

### Preventive behaviour

The majority (124/154; 80.5%) had used chemoprophylaxis before. Eighty-three VFRs (53.9%) had started chemoprophylaxis, 93 (60.4%) had bought chemoprophylaxis and pre-travel advice had been obtained by 104 VFRs (67.5%). Those who had obtained pre-travel advice were more likely to have bought and started chemoprophylaxis (bought: r 0.58, p = <0.01, started:, r 0.60, p = <0.01) (Additional file 2).

### Behavioural determinants

Details regarding behavioural determinants can be found in Additional file 2.

#### Knowledge

Most participants knew malaria was transmitted by mosquitoes, however many misperceptions existed (e g, about transmission via contaminated food and contact with infected people, and about being vaccinated against malaria).

#### Attitudes

A majority of VFRs were not afraid of side effects of tablets and did not think curing malaria is easier than taking preventive tablets. Overall, respondents had faith in malaria tablets and felt that it is bad to use tablets for a long time.

#### Risk perception

Overall, a slight minority of VFRs thought they were immune for malaria. Most participants recognized the risk of infection in the area they were travelling to and that people can die from the disease. However, personal risk was perceived to be lower than the risk of others.

#### Social influence

Most VFRs answered that their friends and/or family used tablets, and that these social contacts encouraged rather than discouraged them to use tablets.

#### Perceived behaviour control

Most participants felt well informed about malaria. Many VFRs thought they could forget a tablet, but most of them did not think the tablet schedule was a difficult regime to follow.

#### Barriers

A minority answered that they had had difficulties with the tablets. Most participants reported to have had enough time to prepare for travel and had to pay for tablets themselves. Swallowing tablets was not regarded a problem by most VFRs.

### Groups at risk

Higher age and travelling for family purposes were positively associated with all behaviours (Block 1; Table 2). Having had malaria and spending more than six weeks in West Africa were negatively associated with starting and buying chemoprophylaxis.

**Table 2:** Logistic regression analyses of determinants predicting prophylactic behaviour

	**Started CP N = 119**				**Bought CP N = 122**				**Obtained pre-travel advice N = 128**			
	**Sig.**	**OR**	**Sig Block**	**Nagelkerke R**	**Sig.**	**OR**	**Sig Block**	**Nagelkerke R**	**Sig.**	**OR**	**Sig Block**	**Nagelkerke R**
			**<0.01**	0.37			<0.01	0.39			**<0.01**	0.22
Age (y)	**0.004**	1.067			**0.005**	1.063			**0.001**	1.067		
Travel purpose	**0.022**	8.906			**0.030**	6.742			**0.006**	7.920		
Previous malaria	**0.028**	0.265			**0.008**	0.210			0.337	0.605		
> 6 weeks in West Africa	**0.001**	0.046			**0.000**	0.064			0.770	0.830		
			**<0.01**	0.55			<0.01	0.52			**<0.01**	0.44
K2 food	-	-			0.514	0.616			-	-		
K3 contact	-	-			0.431	1.875			**0.035**	4.278		
K6 vaccinated	**0.009**	4.467			**0.017**	4.161			0.403	1.522		
A1 afraid	0.460	0.810			-	-			-	-		
A3 faith	0.221	1.824			-	-			-	-		
A4 cure easier than tablets	**0.046**	0.499			**0.023**	0.471			**<0.001**	0.297		
R4 immune	-	-			0.225	0.716			-	-		
S2 encourage	0.867	0.947			0.682	1.137			0.164	1.472		
S3 discourage	0.240	0.608			-	-			-	-		
PBC 1 forgot tablet	0.752	1.091			-	-			-	-		
PBC 2 regime hard	0.090	0.592			-	-			-	-		
			0.14	0.56			0.80	0.52				
E1 used in past	0.139	3.707			0.800	1.214			-	-		
Constant	0.322	0.056			0.641	0.398			.185	.134		

Nagelkerke R = explained variance of the block with included determinants [28].

K2: Determinant Knowledge 2, is malaria transmitted by contaminated food?

K3: Determinant Knowledge 3, is malaria transmitted by infected people?

K6: Determinant Knowledge 6, are you vaccinated against malaria?

A1: Determinant Attitude 1, I am afraid of side effects.

A3: Determinant Attitude 3, I have faith in malaria tablets.

A4: Determinant Attitude 4, it is easier to cure malaria than take tablets.

R4: Determinant Risk perception 4, I am immune for malaria.

S2: Determinant Social 2, my friends/ family encourage use of tables.

S3: Determinant Social 3, my friends/ family discourage use of tablets.

PBC 1: Determinant Perceived Behaviour Control 1, I think I could forget a tablet.

PBC 3: Perceived Behaviour Control 3, do you feel well informed of malaria.

E1: Determinant Previous experience, have you used tablets in the past?

Significant outcomes are indicated in boldface.

### Influential determinants

The more respondents agreed that curing malaria is easier than taking preventive tablets, the less likely they were to have started (OR 0.499, p = 0.046) and bought chemoprophylaxis (OR 0.471, p = 0.023), and to have obtained pre-travel advice (OR 0.297, p = 0.001; Block 2; Table 2). The more convinced respondents were that they had been vaccinated, the more likely they were to have started chemoprophylaxis (OR 4.467, p = 0.009) and to have bought chemoprophylaxis (OR 4.161, p = 0.017).

Respondents who were more convinced that malaria can be transmitted by infected people were more likely to have obtained pre-travel advice (OR 4.278, p = 0.035).

### Previous behaviour

Previous use of chemoprophylaxis did not influence current preventive behaviour (Block 3; Table 2).

### Factors correlating with influential determinants

Correlations of behavioural determinants can be found in Additional file 2. Respondents who felt it is easier to cure than to prevent malaria (A4) more often thought that their friends and family discouraged the use of chemoprophylaxis (S3, r 0.25, p < 0.01). They also less often reported to have faith in malaria tablets (A3, r −0.19, p = 0.02), less often felt well informed about malaria (PBC3, r = −0.20, p = 0.02) and were less likely to have used tablets in the past (E2, r-.26, p < 0.01). Those who thought malaria is transmitted by infected people (K3) more often thought that malaria is transmitted by food (K2, r .66, p < 0.01), that there is a vaccine available (K5, r = .27, p = <0.01,) and that they were vaccinated against malaria (K6, r = 0.32, p < 0.01). They less often felt well informed about malaria (PBC3, r = −0.14, p = 0.09) and reported to have had difficulties with tablets (B1, r = −0.22, p = 0.02) less often.

Respondents who assumed they had been vaccinated (K6) were more likely to incorrectly think a vaccine was available (K5, r = 0.33, p = <0.01), that malaria is transmitted by contaminated food (K2, r = 0.36, p = <0.01) and that malaria is transmitted by infected people (K3, r = 0.32, p = <0.01). They were less likely to think that malaria is a problem in West Africa (R1, r = −0.19, p = 0.02) and had difficulties with tablets less often (B1, r = −0.21, p = 0.02).

## Discussion

### Preventive behaviour and specific risk groups

The study sample performed reasonably well compared to previous reports: 53.9% had started chemoprophylaxis, 60.4% had bought chemoprophylaxis and 67.5% had obtained pre-travel advice. Previously, percentages ranging from 14 to 32% for starting chemoprophylaxis, of 17.6% for buying chemoprophylaxis and of 13.4% for obtaining pre-travel advice have been reported among VFRs [13,17,19,21,22]. However, the fact remains that almost half of the VFRs visited a high-risk destination without adequate protection. Young VFRs, those travelling for longer periods, those travelling for business and those who had had malaria were least likely to use preventive measures against malaria. Those travelling for longer periods [17] and those travelling for business [20,21] have been recognized as risk groups. Previous studies were less conclusive regarding the positive relationship between age and the use of chemoprophylaxis [17,21,22] or taking vaccinations [20]. The previously observed positive relationship between attendance to travel clinics and use of malaria chemoprophylaxis is confirmed with these findings [19-21]. Therefore, increase of attendance of specific risk groups to a travel clinic might be a first step in improving uptake of malaria prophylaxis.

### Determinants - role of attitudes and risk perception

An important determinant that explained preventive behaviour was the opinion that curing malaria is easier than the use preventive tablets. Based on the interviews, this item was included as a measure of VFRs’ general attitude towards prevention of malaria. To properly interpret this determinant however, it should be considered a double-barrelled question, which touches upon two issues that can be modelled according to the PMT [26]. In PMT, behavioural change will occur following threat appraisal (severity of the disease and vulnerability to the disease) if the coping appraisal (self efficacy and response efficacy) is sufficiently high. ‘*Curing the disease is easy*’ may reflect perceived severity of the disease, as one component of perceived risk [26,29]. ‘*Taking tablets is easy*’ may mirror perceived behaviour control (or self efficacy) with regard to taking preventive tablets [24].

The perception of malaria as an easily treatable disease (perceived severity) was, similar to findings described here, negatively related to the uptake of and adherence to malaria prophylaxis in two previous studies [17,18]. Regarding vulnerability (risk perception items), no relationship with uptake of malaria prophylaxis was found. In contrast, the two other studies did find a negative relationship between low perceived personal risk of getting malaria and the use of malaria prophylaxis [17,18]. This apparent incongruence may be explained by the fact that accurate risk perception leads to behaviour change only if both response efficacy and self-efficacy are sufficiently high [26,29].

Previous findings that response efficacy and self-efficacy may be low [18,21], were confirmed by the present study. That is, travellers who had the opinion that it is easier to cure malaria were also less likely to have faith in the effectiveness of malaria tablets (response efficacy), felt less informed about malaria and were less likely to have used tablets in the past (self efficacy).

These results indicate that behaviour change strategies to optimize the use of malaria chemoprophylaxis amongst VFRs should preferably focus on increasing response efficacy (faith in malaria tablets) and additionally pay attention to VFRs’ self efficacy. VFRs who were inclined to think that curing malaria was easier than to use preventive tablets more often felt discouraged to use chemoprophylaxis by their family and friends. Therefore behaviour change strategies could include friends and family members to create a positive social environment. Powerful behaviour change methods in this respect are role modelling and social comparison [30].

### Determinants - role of knowledge and incorrect knowledge

Better knowledge did not improve the use of preventive measures. Surprisingly, VFRs who incorrectly assumed they had been vaccinated against malaria were more likely to have started and bought chemoprophylaxis. This finding is comparable with previously found erroneous beliefs about immunity [17] and the availability of a malaria vaccine [19]. In this study, the incorrect assumption of being vaccinated was associated with other incorrect beliefs, such as that malaria can be transmitted by contaminated food and by infected people.

These findings can be explained in two ways. First, according to the TPB, information accuracy is neither necessary nor sufficient for behaviour change [31]. Actions are determined by subjectively held information (i e, beliefs, either correct or incorrect) rather than by accurate information [31]. Risk reduction strategies should therefore not focus on correcting erroneous knowledge as a purpose in itself. Second, the relationship between incorrectly assuming to be vaccinated and starting chemoprophylaxis might be explained by the fact that during the visit to a pre-travel clinic both a vaccination (against yellow fever) and malaria tablets are provided. Confusion between yellow fever vaccination and malaria prophylaxis in African travellers living in Paris and London has been described [18,19]. Future qualitative research could focus on what exactly happens during pre-travel consultations, to assess whether or not they interfere with the response efficacy and self efficacy-enhancing strategies recommended above.

### Limitations of the study

This study has several limitations. One is the relatively small study population, compromising statistical analysis. Due to the fact that more than half the VFRs were transit passengers, country of residence varied. Possibly, attitudes about prevention vary between those coming from Central/North America and Europe. Also, this cross-sectional study was merely a snapshot of the situation without a longitudinal follow-up regarding adherence to prophylaxis regimes. It should be noted that it was not checked whether participants carried the tablets they reported. As confusion about preventive drugs among travellers has been described (e g, paracetamol was mistakenly reported as a malaria preventive drug [20]), the percentages of having bought and started chemoprophylaxis may be too optimistic. Because of time constraints the number of questions in the questionnaire had to be minimized. Internally consistent determinant scales could not be constructed. This indicates that the beliefs measured did not entirely cover the theoretical determinants under consideration. Thanks to the preceding qualitative enquiry, however, the questionnaire presumably included the most salient beliefs. Age was included in the model instead of migration time. This may be debatable as information for public health measures may be lost; however, as pre-travel advice is provided in a clinical setting this variable was preferred. Finally, as not all participants fully mastered the English language, some questions might have been misunderstood.

## Conclusion

This study population performed relatively well compared to other VFR populations as more than half had started chemoprophylaxis. However, improvements remain necessary and prevention strategies should focus on young travellers, business travellers, long-term travellers and those who have previously experienced malaria. Pre-travel consultations should not aim to correct erroneous beliefs about malaria as such. Preventive strategies should focus on increasing response efficacy (e g, the effectiveness of malaria prophylaxis) and self-efficacy (related to the complexity of the medication regime). Such strategies could be strengthened by including friends and family members to create the positive social environment needed to further improve the use of malaria prophylaxis.

## Competing interests

The authors have declared that they have no competing interests.

## Authors’ contributions

RW collected and analysed the study data and wrote the first draft of the manuscript. JH participated in the study design, supervised statistical analyses and critically revised the manuscript. PB conceived the study, collected the study data, participated in its coordination and contributed to drafting the manuscript. MPG contributed to study design and coordination and contributed to the drafting of the manuscript. MvV participated in the study design and the coordination of the study and contributed to the drafting of the manuscript. All authors have contributed to and approved the manuscript’s final version.

## Supplementary Material

Additional file 1 Questionnaire. Click here for file

Additional file 2 Correlations between determinants influencing behaviour. Click here for file
