# Supplementary material for: Towards improved uptake of malaria chemoprophylaxis among West African travellers: identification of behavioural determinants
Source: Malar J. 2013 Oct 10;12:360. doi: 10.1186/1475-2875-12-360 (PMC3852732; doi:10.1186/1475-2875-12-360)
Supplement: Additional file 1 — Questionnaire. [file 1475-2875-12-360-S1.docx]

Additional file 1 Questionnaire

| Entered by interviewer: male or female |
| --- |
| Demographics |
| 1. What is your age? |
| 2. Where are you born? |
| 3. Where are your parents born? |
| 4. How long have you been living outside West Africa? (if answer to question 2. was West Africa) |
| 5. Have you ever been back to your country of birth? (or the country of origin of your parents?)   - Yes, 1-3 times - Yes, 4-6 times - Yes, >6 times - No |
| 6. Are you going to Ghana now?   - Yes - No - I don’t want to say |
| 7. What is the duration of this trip? |
| 8. What is the purpose of this trip?   - Visiting friends and relatives - Funeral/Wedding/ family affairs - Business - Holiday - Other - I don’t want to say |
| 9. Did you receive pre-travel advice at a healthcare center?   - Yes - No - I don’t want to say |
| Determinants |
| 10. B2 have you had enough time to prepare travel?   - Yes - No - I don’t want to say |
| 11. O2 have you bought malaria tablets?   - Malarone - Lariam - Other - No - No, forgotten to buy - No, I will buy them at the destination - No, because it is to expensive - I don’t want to say |
| 12. B3 do you have to pay for pre-travel advice and/or tablets yourself?   - Yes, only for pre-travel advise - Yes, only for malaria tablets - Yes, for both - No - No, I get both (partly) refunded - No, I get pre-travel advise (partly) refunded - No, I get malaria tablets (partly) refunded - I don’t know - I don’t want to say |
| 13. O1 have you already started with anti-malaria tablets?   - Yes - Yes, but delayed - Yes, but stopped because of side effects - No - I don’t want to say |
| 14. If no, why not? |
| 15. E1 Have you used tablets in the past?   - Yes - No - I don’t want to say |
| 16. B1 have you had difficulties with tablets?   - I have had side effects - I did not use all tablets - I did not have any problems - I don’t want to say |
| 17. Have you ever had malaria?   - Yes, before I came to the Netherlands - Yes, during a journey before - No - I don’t know - I don’t want to say |
| 18. K6 are you vaccinated against malaria?   - Yes - No - I don’t know |
| 19. R1 do you think malaria is a problem in West Africa?   - Malaria is a serious problem in West Africa. - There is a low risk of malaria in West Africa. - There is no malaria in West Africa. |
| 20. R2 do you think you can die of Malaria?   - Yes - No - Only weak people die from malaria - I don’t know |
| 21. A4 it is easier to cure malaria than to take anti-malaria tablets   - I totally agree - I agree - Neutral - I disagree - I totally disagree |
| 22. R3 my personal risk is lower than the risk of others   - I totally agree - I agree - Neutral - I disagree - I totally disagree |
| 23. R4 i am immune for malaria   - I totally agree - I agree - Neutral - I disagree - I totally disagree |
| 24. B4 it is a problem to swallow tablets   - I totally agree - I agree - Neutral - I disagree - I totally disagree |
| 25. A1 i am afraid of side effects of anti-malaria tablets   - I totally agree - I agree - Neutral - I disagree - I totally disagree |
| 26. A2 it is bad to use tablets for a long time   - I totally agree - I agree - Neutral - I disagree - I totally disagree |
| 27. A3 i have faith in the working of anti-malaria tablets   - I totally agree - I agree - Neutral - I disagree - I totally disagree |
| 28. PBC 1 i think I could forget a tablet   - I totally agree - I agree - Neutral - I disagree - I totally disagree |
| 29. PBC 2 i think the regime is difficult   - I totally agree - I agree - Neutral - I disagree - I totally disagree |
| 30. S1 my friends/ family use anti-malaria tablets   - I totally agree - I agree - Neutral - I disagree - I totally disagree |
| 31. S2 my friends/ family encourage the use of tables ^*^   - I totally agree - I agree - Neutral - I disagree - I totally disagree |
| 32. S3 my friends/ family discourage use of tablets ^*^   - I totally agree - I agree - Neutral - I disagree - I totally disagree |
| 33. K1 do mosquitoes transmit malaria?   - Yes - No - I don’t know |
| 34. K2 is malaria transmitted by contaminated food?   - Yes - No - I don’t know |
| 35. K3 is malaria transmitted by contact with an infected person?   - Yes - No - I don’t know |
| 36. K4 can you recover without medicine?   - Yes - No - I don’t know |
| 37. PBC 3 do you feel well informed of malaria?   - Yes - No |
| 38. K5 is there a vaccine against malaria?   - Yes - No - I don’t know |

K 1-6: Determinant Knowledge 1-6

A 1-4: Determinant Attitude 1-4,

R 1-4: Determinant Risk perception 1-4

S 1-3: Determinant Social 1-3

PBC 1-3: Determinant Perceived Behaviour Control 1-3

E 1: Determinant Previous experience 1

O 1-3: Outcome 1-3
