# Supplementary material for: Towards improved uptake of malaria chemoprophylaxis among West African travellers: identification of behavioural determinants
Source: Malar J. 2013 Oct 10;12:360. doi: 10.1186/1475-2875-12-360 (PMC3852732; doi:10.1186/1475-2875-12-360)
Supplement: Additional file 2 — Correlations between determinants influencing behaviour. [file 1475-2875-12-360-S2.docx]

**Additional file 2 Correlations between determinants influencing behaviour**

|  |  | Mean | SD | N (%) |  | O1 | O2 | O3 | E1 | K1 | K2 | K3 | K4 | K5 | K6 | A1 | A2 | A3 | A4 | R1 | R2 | R3 | R4 | S1 | S2 | S3 | P1 | P2 | P3 | B1 | B2 | B3 | B4 |
| --- | --- | --- | --- | --- | --- | --- | --- | --- | --- | --- | --- | --- | --- | --- | --- | --- | --- | --- | --- | --- | --- | --- | --- | --- | --- | --- | --- | --- | --- | --- | --- | --- | --- |
| Outcome | O1 Have you started using tablets? ^a^ |  |  | 83/154  (53.9) | Corr  Sig | 1 |  |  |  |  |  |  |  |  |  |  |  |  |  |  |  |  |  |  |  |  |  |  |  |  |  |  |  |
|  | O2 Have you bought tablets? ^a^ |  |  | 93/154  (60.4) | Corr  Sig | .88*  <.01 | 1 |  |  |  |  |  |  |  |  |  |  |  |  |  |  |  |  |  |  |  |  |  |  |  |  |  |  |
|  | O3 Did you obtain pre-travel advice? ^a^ |  |  | 104/154  (67.5) | Corr  Sig | .58*  <.01 | .60*  <.01 | 1 |  |  |  |  |  |  |  |  |  |  |  |  |  |  |  |  |  |  |  |  |  |  |  |  |  |
| E* | E1 Used CP in past ^a^ |  |  | 124/146  (84.9) | Corr  Sig | .27*  <.01 | .17*  .04 | .04  .60 | 1 |  |  |  |  |  |  |  |  |  |  |  |  |  |  |  |  |  |  |  |  |  |  |  |  |
| Knowledge | K1 Is malaria transmitted by mosquitoes? ^b^ |  |  | 151/154  (98.1) | Corr  Sig | .15  .06 | .08  .34 | .00  .97 | .12^*^  .17 | 11 |  |  |  |  |  |  |  |  |  |  |  |  |  |  |  |  |  |  |  |  |  |  |  |
|  | K2 Is malaria transmitted by contaminated food? ^c^ |  |  | 59/153  (38.6) | Corr  Sig | .12  .15 | .15  .06 | .12  .13 | -.09  .27 | -.08  .32 | 1 |  |  |  |  |  |  |  |  |  |  |  |  |  |  |  |  |  |  |  |  |  |  |
|  | K3 Is malaria transmitted by infected people? ^c^ |  |  | 40/153  (26.0) | Corr  Sig | .11  .19 | .18*  .03 | .22*  <.01 | -.05  .52 | -.02  .78 | .66*  <.01 | 1 |  |  |  |  |  |  |  |  |  |  |  |  |  |  |  |  |  |  |  |  |  |
|  | K4 Can you recover without medicine? ^c^ |  |  | 50/153  (32.7) | Corr  Sig | -.02  .79 | .00  .98 | -.08  .33 | .00  .99 | -.20  .01 | -.07  .42 | -.00  .98 | 1 |  |  |  |  |  |  |  |  |  |  |  |  |  |  |  |  |  |  |  |  |
|  | K5 Is there a vaccine against malaria?^c^ |  |  | 119/152  (77.3) | Corr  Sig | .00  .94 | .03  .76 | .11  .19 | .01  .95 | -.07  .36 | .22*  <.01 | .27  <.01 | -.01  .88 | 1 |  |  |  |  |  |  |  |  |  |  |  |  |  |  |  |  |  |  |  |
|  | K6 Are you vaccinated against malaria?^c^ |  |  | 86/154  (55.8) | Corr  Sig | .23*  <.01 | .27*  <.01 | .25*  <.01 | .11  .19 | .06  .43 | .36*  <.01 | .32*  <.01 | -.13  .10 | .33**  <.01 | 1 |  |  |  |  |  |  |  |  |  |  |  |  |  |  |  |  |  |  |
| Attitude | A1 I am afraid of side effects ^d^ | 2.56 | .98 |  | Corr  Sig | -.15*  .07 | -.10  .20 | -.04  .63 | -.11  .19 | -.21*  .01 | -.03  .76 | .03  .74 | .23*  <.01 | -.02  .84 | -.04  .67 | 1 |  |  |  |  |  |  |  |  |  |  |  |  |  |  |  |  |  |
|  | A2 It is bad to use tablets for a long time ^d^ | 3.23 | .93 |  | Corr  Sig | -.02  .81 | -.07  .40 | -.07  .43 | .03  .70 | -.02  .85 | -.03  .74 | -.04  .64 | .08  .33 | .18  .03 | -.02  .85 | .08  .34 | 1 |  |  |  |  |  |  |  |  |  |  |  |  |  |  |  |  |
|  | A3 I have faith in malaria tablets ^d^ | 3.86 | .64 |  | Corr  Sig | .20*  .01 | .13  .11 | -.00  .97 | .14  .10 | .19  .02 | -.01  .89 | -.12  .13 | -.13  .12 | -.04  .59 | -.08  .35 | -.22*  <.01 | .11  .17 | 1 |  |  |  |  |  |  |  |  |  |  |  |  |  |  |  |
|  | A4 It is easier to cure malaria than take tablets ^d^ | 2.40 | 1.03 |  | Corr  Sig | -.34*  <.01 | -.30*  <.01 | -.31*  <.01 | -.26*  <.01 | -.12  .15 | .07  .36 | .09  .30 | .08  .36 | .04  .57 | -.04  .64 | .12  .16 | -.02  .85 | -.19  .02 | 1 |  |  |  |  |  |  |  |  |  |  |  |  |  |  |
| Risk Perception | R1 Do you think malaria is a problem in W-A? ^e^ |  |  | 105/154  (68.2) | Corr  Sig | -.02  .84 | -.10  .23 | .00  .97 | .04  .65 | .11*  .19 | -.30*  .00 | -.21  .01 | -.04  .63 | -.18  .03 | .-19*  .02 | .10  .24 | .01  .93 | .07  .42 | -.10  .22 | 1 |  |  |  |  |  |  |  |  |  |  |  |  |  |
|  | R2 Do you think you can die of Malaria? ^f^ |  |  | 142/146  (97.3) | Corr  Sig | .01  .89 | .03  .71 | -.03  .72 | -.07  .39 | -.02  .77 | -.04  .60 | -.01  .94 | .03  .75 | .11  .19 | -.07  .38 | .09  .26 | .13  .12 | .09  .27 | -.04  .62 | .26*  <.01 | 1 |  |  |  |  |  |  |  |  |  |  |  |  |
|  | R3 My personal risk is lower than that of others ^d^ | 3.43 | .94 |  | Corr  Sig | .05  .50 | .02  .79 | .09  .30 | .09  .29 | .12  .15 | -.15  .07 | -.03  .73 | -.09  .25 | .08  .32 | -.05  .56 | -.02  .80 | -.07  .43 | .10  .20 | .05  .58 | .06  .43 | -.10  .22 | 1 |  |  |  |  |  |  |  |  |  |  |  |
|  | R4 I am immune for malaria ^d^ | 2.77 | .96 |  | Corr  Sig | -.11  .17 | -.17*  .04 | -.02  .81 | -.09  .29 | -.13  .10 | .04  .59 | -.03  .69 | .05  .58 | .20  .02 | .12  .15 | .07  .40 | .09  .27 | -.13  .12 | .18*  .03 | -.06  .46 | .13  .11 | .08  .32 | 1 |  |  |  |  |  |  |  |  |  |  |
| Social | S1 My friends/ family use tablets ^d^ | 3.79 | .59 |  | Corr  Sig | .06  .45 | .09  .28 | -.04  .62 | .11  .20 | .03  .73 | -.07  .36 | -.03  .67 | -.03  .73 | -.11  .19 | .04  .67 | .05  .52 | -.16  .05 | .01  .95 | .07  .36 | .13  .11 | -.06  .46 | .20*  .01 | .09  .29 | 1 |  |  |  |  |  |  |  |  |  |
|  | S2 My friends/ family encourage use of tables ^d^ | 3.50 | .82 |  | Corr  Sig | .16*  .04 | .22*  <.01 | .22*  <.01 | .03  .69 | .15  .07 | .09  .27 | .06  .50 | -.12  .14 | -.09  .27 | .07  .40 | .08  .35 | -.04  .66 | .05  .53 | -.11  .18 | .03  .73 | -.10  .22 | -.00  .98 | -.18  .03 | .35*  <.01 | 1 |  |  |  |  |  |  |  |  |
|  | S3 My friends/ family discourage use of tablets ^d^ | 2.36 | .67 |  | Corr  Sig | -.20*  .01 | -.08  .35 | .05  .58 | -.11  .19 | -.06  .43 | -.05  .53 | .07  .36 | .18*  .03 | .17  .04 | .03  .68 | .06  .44 | -.17*  .04 | -.12  .14 | .25*  <.01 | -.03  .76 | .09  .26 | .03  .74 | .03  .74 | -.07  .42 | -.09  .29 | 1 |  |  |  |  |  |  |  |
| PBC | PBC 1 I think I could forget a tablet ^d^ | 3.07 | 1.0 |  | Corr  Sig | -.14*  .10 | -.10  .24 | -.10  .22 | -.04  .61 | -.04  .67 | -.12  .14 | -.03  .74 | .09  .28 | -.03  .73 | -.06  .46 | .22*  <.01 | -.10  .21 | .01  .94 | .07  .39 | .11  .19 | .13  .12 | -.09  .30 | -.09  .28 | .08  .31 | .13  .11 | .13  .10 | 1 |  |  |  |  |  |  |
|  | PBC 2 I think the regime is difficult ^d^ | 2.71 | .95 |  | Corr  Sig | -.14*  .08 | -.06  .44 | .01  .92 | -.03  .70 | -.14  .08 | .05  .59 | .01  .89 | .10  .23 | .00  .98 | .03  .72 | .13  .11 | -.07  .38 | -.14  .09 | .08  .34 | .04  .64 | .08  .33 | .07  .37 | -.03  .74 | .06  .45 | -.02  .83 | .07  .40 | .28*  <.01 | 1 |  |  |  |  |  |
|  | PBC 3 Do you feel well informed of malaria? ^a^ |  |  | 131/153  (85.6) | Corr  Sig | .03  .72 | -.07  .41 | -.01  .93 | .20*  .02 | .08  .35 | -.21  .01 | -.14  .09 | .05  .56 | -.13  .12 | -.03  .72 | -.03  .68 | .10  .20 | .05  .51 | -.20*  .02 | .21*  .01 | .18*  .03 | -.04  .59 | .06  .46 | .12  .14 | .08  .36 | -.03  .75 | -.06  .45 | -.08  .33 | 1 |  |  |  |  |
| Barriers | B1 Have you had difficulties with tablets? ^a^ |  |  | 15/ 120  (12.5) | Corr  Sig | -.05  .58 | -.09  .32 | -.02  .82 | .00 | --  -- | -.19*  .04 | -.22*  .02 | .07  .48 | -.16  .09 | -.21*  .02 | .23*  .01 | -.10  .30 | -.04  .65 | -.06*  .51 | .20  .03 | .08  .43 | .01  .94 | .06  .49 | -.02  .86 | -.20*  .03 | -.04  .68 | .12  .21 | .09  .31 | -.09  .36 | 1 |  |  |  |
|  | B2 Have you had enough time to prepare travel? ^a^ |  |  | 129/152  (84.9) | Corr  Sig | .09  .25 | .11  .18 | .10  .24 | -.12  .15 | -.06  .46 | .11  .17 | .12  .13 | -.06  .46 | .00  .97 | .07  .40 | .09  .28 | -.06  .46 | -.09  .26 | .14  .10 | -.05  .54 | -.07  .40 | .05  .58 | .17*  .03 | -.09  .28 | -.12  .14 | -.05  .58 | -.09  .26 | -.09  .28 | .04  .60 | .04  .64 | 1 |  |  |
|  | B3 Do you have to pay for tablets yourself? ^a^ |  |  | 86/ 142  (60.6) | Corr  Sig | .05  .55 | .02  .81 | .05  .54 | ..14  .11 | .15  .08 | -.21*  .01 | -.16  .07 | .00  .98 | .05  .55 | -.08  .36 | .11  .21 | -.00  .98 | .01  .94 | -.04  .62 | .23*  .01 | .08  .34 | .18*  .04 | -.15  .08 | -.04  .68 | -.06  .46 | .01  .90 | .09  .27 | .12  .17 | .01  .92 | .02  .82 | -.07  .42 | 1 |  |
|  | B4 Is it a problem to swallow tablets? ^d^ | 2.19 | .81 |  | Corr  Sig | .01  .94 | .04  .61 | -.06  .45 | .00  .99 | -.32**  .00 | .04  .65 | .01  .88 | .14  .09 | .06  .49 | -.02  .81 | .25*  <.01 | -.06  .47 | -.16*  .04 | .14  .09 | -.05  .55 | .04  .66 | -.09  .26 | -.03  .72 | -.05  .53 | -.07  .42 | -.04  .60 | .14  .09 | .08  .31 | -.21*  .01 | .12  .21 | .15  .07 | -.20  .02 | 1 |
|  | * E = previous experience; ^a^ 1 = Yes, 0 = No, 99 = don’t want to say; ^b^ 1= yes 0 = no/don’t know; ^c^ 1 = yes/don’t know, 0 = no; ^d^ 1= totally disagree, 2 = disagree, 3 = neutral, 4= agree, 5 = totally agree; ^e^ 1= there is a high risk, 0= there is a low risk/there is no risk; ^f^ 1= yes/only weak people die from malaria, 0= no, SD = Standard deviation. Mean and SD are provided for variables with a scale outcome, N(%) = is the number and percentage of correct answers. | | | | | | | | | | | | | | | | | | | | | | | | | | | | | | | | |

**Additional file 2 Correlations with outcomes**

Determinants that correlated positively with the outcome ‘started’ were the assumption of having been vaccinated against malaria, having faith in the working of the tablets and whether friends and family encouraged using tablets. A negative correlation was found if VFRs reported to be afraid of side effects, if they thought it was easier to cure malaria than to use preventive measures, if they felt their friends and family discouraged the use of chemoprophylaxis, if they thought they would forget a tablet or if they thought the regime was difficult. Determinants that correlated positively with the outcome ‘tablets bought’ were the assumption that malaria is transmitted by food or by contact with infected people, that they had been vaccinated and that friends and family encouraged the use of chemoprophylaxis. The assumption that curing malaria is easier than taking preventive tablets and presumed immunity against malaria correlated negatively with buying tablets. Determinants that correlated positively with obtaining pre-travel advice were the assumption that malaria is transmitted by contact with infected people, presumed vaccination against malaria and encouragement of using chemoprophylaxis by friends and family. The assumption that curing malaria is easier than taking preventive tablets correlated negatively with obtaining pre-travel advice
